# An antenna model for the Purcell effect

**DOI:** 10.1038/srep12956

**Published:** 2015-08-10

**Authors:** Alexander E. Krasnok, Alexey P. Slobozhanyuk, Constantin R. Simovski, Sergei A. Tretyakov, Alexander N. Poddubny, Andrey E. Miroshnichenko, Yuri S. Kivshar, Pavel A. Belov

**Affiliations:** 1 ITMO University, St. Petersburg 197101, Russia; 2 Nonlinear Physics Center, Research School of Physics and Engineering, Australian National University, Canberra ACT 0200, Australia; 3 Aalto University, School of Electrical Engineering, Aalto FI-00076, Finland; 4 Ioffe Physical-Technical Institute of the Russian Academy of Sciences, St. Petersburg, 194021, Russia

## Abstract

The Purcell effect is defined as a modification of the spontaneous emission rate of a quantum emitter at the presence of a resonant cavity. However, a change of the emission rate of an emitter caused by an environment has a classical counterpart. Any small antenna tuned to a resonance can be described as an oscillator with radiative losses, and the effect of the environment on its radiation can be modeled and measured in terms of the antenna radiation resistance, similar to a quantum emitter. We exploit this analogue behavior to develop a general approach for calculating the Purcell factors of different systems and various frequency ranges including both electric and magnetic Purcell factors. Our approach is illustrated by a general equivalent scheme, and it allows resenting the Purcell factor through the continuous radiation of a small antenna at the presence of an electromagnetic environment.

The Purcell effect is defined as a modification of the spontaneous emission lifetime of a quantum source induced by its interaction with environment^1^,^2^,^3^,^4^,^5^,^6^,^7^,^8^,^9^, and it was first described by E.M. Purcell^1^ in 1946 in the context of nuclear magnetic resonance. At present, this effect is widely used in microcavity light-emitting devices^10^,^11^,^12^, single-molecule optical microscopy^13^,^14^, microscopy of single NV centers in nanodiamonds^15^ and Eu^3+^-doped nanocrystals^9^, and for visualization of biological processes with participation of large molecules such as DNA^16^ (for a comprehensive review, see^17^).

Here, we analyze both theoretically and experimentally a classical counterpart of the Purcell effect for subwavelength electric and magnetic dipole antennas. We generalize the approach employed in nanophotonics to the case of microwave antennas and recover the well-known expression for the Purcell factor in the context of equivalent circuit model that is elegant due to its inherent simplicity and excellent agreement with existing models. Using this result, we propose a new method to measure directly the Purcell factor through the input impedance of a small antenna, and verify this approach experimentally, generalizing the results of Refs 17, 18, 19.

First, we provide a brief overview of several equivalent definitions of the Purcell factor—a value which describes this effect quantitatively—and the existing theoretical and experimental approaches. The notion of the Purcell effect is based on the quantum electrodynamics describing weak coupling of an emitter and a resonating object (e.g., nanoantenna^17^ or optical cavity^10^,^11^,^12^). The weak and strong coupling regimes^20^,^21^ can be distinguished by comparing the so-called emitter-field coupling constant 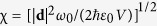 with the decay rate of a photon in a cavity γ and the nonradiative decay rate of the excited state γ_dis_. Here, *ω*_0_ and **d***** ***= *e*〈2|**r**|1〉 are the frequency of the excited-to-ground state transition (2 → 1) and its dipole moment, respectively, *e* is the electron charge, *V* is the effective volume of the resonator mode, *ε*_0_ is the vacuum permittivity. Hereinafter, we employ the SI units, the equivalent expressions in the CGS units can be restored by replacing *ε*_0_ by 1/(4*π*).

In the weak-coupling regime when 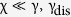, the hybridization of the quantum emitter and resonator eigenstates is weak. Therefore, the frequency *ω*_0_ of the spontaneous emission is not modified by the resonator, i.e., the Lamb-shift is significantly small compared to the original resonant frequency, and the light-matter interaction leads to a modification of the decay rate only. The dipole moment of the optical transition **d** and its classical counterpart, dipole moment **d**_1_ remain unperturbed and the dipole moment in quantum and classical cases are related via **d**_1_ = 2**d**(see e.g. Ref. 22, p.250). A ratio of the decay rate γ in the vicinity of the resonator to the decay rate of the same emitter in free space γ_0_ can be written as^20^:


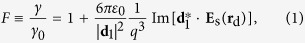


where 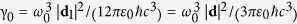
20, *q* = *ω*/*c* is wavenumber in free space, **E**_s_(**r**_d_) is the scattering part of electric field evaluated at the quantum emitter position **r**_d_, and the quantum emitter has a dipole moment **d**_1_ oscillating at the frequency *ω*_0_. The quantity *F* is called the Purcell factor. According to Eq. (1), the magnitude of the Purcell factor does not depend on the magnitude of the transition dipole moment **d**, because the scattered field value is directly proportional to the dipole moment, thus the numerator and denominator are free of **d**. It is important to note that Eq. 1 can be applied to any arbitrary electromagnetic environment of the emitter different from free space^17^. Moreover, the concept of Purcell’s factor can be extended to optical emitters which cannot be modeled as a point electric dipole^23^. The Purcell factor can be also understood in terms of the local density of photonic states modified by the presence of the object^24^.

The above expression for γ_0_ does not take into account the non-radiative decay (it is assumed that 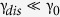) and results from the standard formula for the power radiated by a Hertzian dipole **d**_1_ at frequency *ω*_0_:


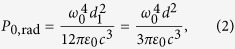


namely,


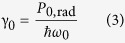


is the ratio of *P*_0,rad_ to the photon energy. In the weak coupling regime, the environment modifies only the radiated (far-zone) power and the dissipation of power in the volume outside of the emitter. Thus, the decay factor modified by the environment can be written as


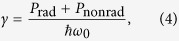


thus the Purcell factor can be expressed also as


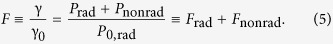


Here, *P*_rad_ is the power radiated in the far zone (enhanced by the environment) and *P*_nonrad_ is the power dissipated in the environment.

If the electromagnetic environment is lossless, the last term vanishes and the Purcell factor describes the change of the total radiated power *P*_rad_ at the frequency of the emitter:


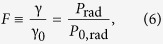


where the subscript 0 refers to the corresponding value for the same emitter in free space. If the emitter is located in a lossy medium (perhaps inhomogeneous) the Purcell factor Eq. (1) has two contributions: (i) the far-field emission, (ii) the Joule losses in the environment^25^. When the environment is described by position-dependent dielectric constant *ε*(**r**′), the Joule loss contribution to the Purcell factor can be presented as^26^:


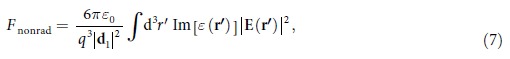


where **E**(**r**′) is the total field produced by the dipole **d**_1_ at the point **r**′ which is integrated over the entire surrounding space.

Here, one may introduce the radiation efficiency of the quantum source *ξ* in the same way as it is usually done in the antenna theory^27^: *ξ* 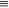 *F*_rad_/*F* = (*F* − *F*_nonrad_)/*F*. The total quantum yield *Q* of the emitter is determined by the competition between the far-field radiation, the Joule losses, and the internal non-radiative losses of the emitter γ_dis_:


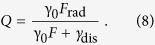


We notice that in Eq. (6), the decay rate of an emitter in free space is assumed to be γ_0_, i.e. nonradiative losses inside the emitter are neglected. This is a realistic approximation for many quantum dots and fluorescent dye molecules (e.g. in^28^ γ_dis_ and γ_0_ were separately measured for nanocrystal quantum dots, and it was shown that 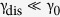).

For quantum emitters the total Purcell factor is measured either directly by evaluating the speedup of the time-resolved photoluminescence^8^ or indirectly, for example, using the Raman spectroscopy^29^. Large values of *F* can be achieved with nanoantennas — resonant devices that effectively convert the near field of quantum sources to propagating optical radiation^30^,^31^. This transformation is carried out by means of impedance matching between the quantum source and the nanoantenna^19^,^32^,^33^. Another possibility to attain significantly large values of the Purcell factor is provided by hyperbolic metamaterials (see the review^8^).

## Retrieval of the Purcell factor through an input impedance

### General approach

**Figure 1:**
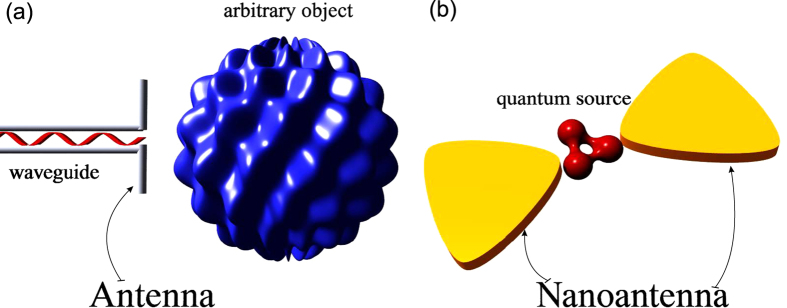
The classical (**a**) and quantum (**b**) realizations of the Purcell effect.

Let us consider an arbitrary radiating electric dipole with the dipole moment d at presence of an arbitrary passive object, as shown in Fig. 1(a). We attribute number “1” to the dipole and number “2” to the object. The total electric field created by the dipole “1” at its origin **E**_1_(**r**_*d*_) can be decomposed into two parts, i.e., **E**_1_(**r**_*d*_) = **E**_11_(**r**_*d*_) + **E**_12_(**r**_*d*_), where **E**_11_(**r**_*d*_) is the field created by the dipole “1” in the absence of the object “2” and **E**_12_(**r**_*d*_) 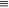 **E**_s_(**r**_*d*_) is the field scattered by the object. The total power delivered by the radiating particle to the environment reads


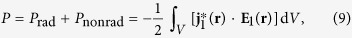


where *V* is the volume of the radiating dipole and 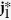 is the electric current density in that volume. Thus, it splits into two parts *P* = *P*_11_ + *P*_12_, where *P*_11_ is the power radiated by the same dipole in the absence of object “2” (the same as *P*_0,rad_ in Eq. (2), and


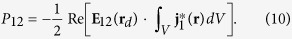


Here we assume that the radiating dipole “1” has a sufficiently small volume, such that the spatial variation of field **E**_12_ over *V* can be neglected. Since the classical electric dipole moment is defined via the electric current density **j**_1_ as


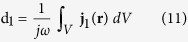


for the time dependence in the form exp(*jωt*), Eq. (10) can also be rewritten as


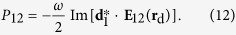


After substitution of Eq. (2) into Eq. (5) yields:


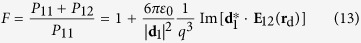


i.e. the well-known result Eq. (1), which is applicable to both classical and quantum emitters (weakly coupled to an arbitrary object).

The last result can be rewritten in terms of the input impedances and Green’s function. First, by the definition of radiation resistance we can write:


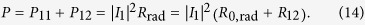


Here the effective current *I*_1_ referred to the origin **r**_*d*_ is related with the dipole moment as *I*_1_ = *jωd*_1_/*l*_1_, *l*_1_ is the effective length of the dipole 1. The radiation resistance of an optically small particle with the effective length *l* reads as^27^:


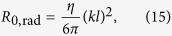


where 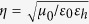 and 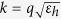 are the wave impedance and the wave number of the host medium, respectively. The additional (mutual) resistance *R*_12_ = Re *Z*_12_ caused by the field scattered from the radiation-enhancing object *E*_12_ can be found separately. This is an interesting and relevant problem which will be studied in the next subsection.

Eq. (14), rewritten as *F* = *P*/*P*_11_ = *R*_rad_/*R*_0,rad_, may already serve as an alternative to the commonly used expression Eq. (1). From the general theory of antennas it is well-known that the input resistance of a short dipole is equal to the radiation resistance, when the dissipative losses inside the antenna are neglected^27^. Thus, if our emitter is low-loss 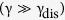, we can write an equivalent relation for the Purcell factor of an arbitrary object (inhomogeneous environment) for a low-loss emitter (valid for both quantum or classical emitters):


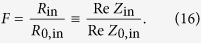


In some situations it may be more convenient to measure or calculate the input impedances (*Z*_0,in_ and *Z*_in_) of the emitter “1” in the absence and presence of object “2” than to accurately find the scattered field. Then Eq. (16) allows direct measurement/calculation of the Purcell factor through the real parts of these impedances. Thus, Purcell factor for a dipole emitter is alternatively determined by the modification of the resistive part of its input impedance. Non-radiative losses in the above formula are lumped inside the *R*_in_ since the additional resistance *R*_12_ is not purely radiative. Mutual coupling effectively brings the losses of the object “2” into emitter “1”.

Let us now show that Eq. (16) fits into another known representation of the Purcell’s factor—through Green’s function^34^. The electric field produced by a dipole **d**_1_ stretched along the *z*-axis is related to the dyadic Green’s function of an inhomogeneous environment 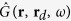 as follows^20^:


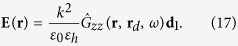


In order to relate the Green’s function to the input impedance *Z*_in_ of our dipole “1” we use the Brillouin method of induced electromotive forces (IEMF)^27^:


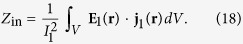


The expression in the numerator is called IEMF in radio science, and *I*_1_ in the denominator is the current though the central cross section of the dipole. In the short antenna approximation, i.e., *kl*_1_ → 0 the result reduces to^27^:


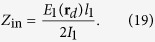


The above expression establishes a relationship between the magnitude of the input impedance of a short dipole and the value of the *total* (not just the scattered part) electric field **E**_1_ = **E**_1_*z*_0_ at the dipole origin. Now, recall the definition of the Green’s function in Eq. (17) and using the relation *I*_1_*l*_1_ = *jωd*_1_ we obtain an expression that links the Green’s function with the input impedance of the dipole 1: 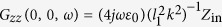. Now we may rewrite Eq. (16) in form


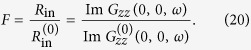


This expression is equivalent to Eq. (2.4) from^34^.

Thus, it is possible to find the Purcell factor either using the standard techniques, such as Eqs (1), (20), or using Eq. (16), in terms of input resistances.

### Equivalent circuit for the Purcell factor

Here we explain how to find the input resistance *R*_in_ of an optical (e.g. fluorescent) emitter at the presence of an optically small resonator. Such resonators, called *nanoantennas,* are used to enhance the spontaneous emission of isolated quantum emitters (see Refs 17,30,33). Though this treatment is focused on quantum emitters, our consideration is fully classical and the approach towards the conclusions are based on the concept of electromagnetically coupled oscillators. Therefore, it is relevant to illustrate our approach by equivalent circuits.

For instance, we notice that a quantum emitter and a nanoantenna (which is a classical resonant scatterer), both of these objects, in the absence of tunneling effects interact purely electromagnetically, and their coupling is governed by Maxwell’s equations. Therefore, both of them can be described in terms of resonant *RLC*-circuits. An attempt to build such schemes has been made in work^19^, however without relevant practical results. In the present paper, we introduce an alternative equivalent circuit for radiating systems comprising an optical emitter and a nanoantenna. This circuit model illustrates a simple algorithm for calculating the additional term *R*_12_ is incorporated into the expression of the input resistance *R*_in_ in presence of object “2”. This term is designated as mutual resistance *R*_*m*_ 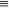 *R*_12_.

**Figure 2:**
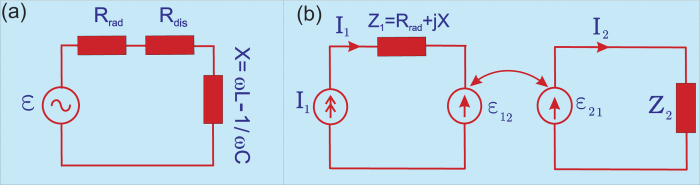
(**a**) An equivalent scheme of a resonant dipole scatterer. (**b**) Equivalent schemes of an emitter and a nanoantenna in terms of induced electromotive forces.

First, we recall the well-known circuit model of an optically small dipole scatterer excited by an external electric field **E** = **z**_0_*E* (see Ref. 35). The current that is induced in a short dipole antenna of effective length *l* reads as *I* = *El*/*Z*, where *Z* = *R*_rad_ + *R*_dis_ + *jX* is the total impedance of the particle, see Fig. 2(a). Here we have split Re(*Z*) into the radiation resistance *R*_rad_, and dissipation resistance *R*_dis_. Since the induced dipole moment equals *d*_ind_ = *Il*/*jω* (assume for simplicity that **d**_ind_ = **z**_0_*d*_ind_ that holds for a spherical particle for any polarization and for an ellipsoidal one polarized along one of its axes), the inverse polarizability *α*^−1^ 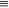 *E*/*d*_ind_ reads as


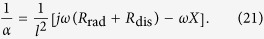


Substituting Eq. (15) into Eq. (21), we find


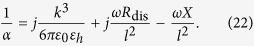


This is the well-know formula for the polarizability of a lossy dipole scatterer which is applicable to both quantum emitter and nanoantenna. However, in this paper we neglect the induced part of the dipole moment of the quantum emitter as well as the hybridization of its states. In the weak coupling regime **d**_1_(*ω*_0_) = 2**d**. But, the emission spectrum has the Lorentzian shape^20^, and this implies that we have to consider the polarization of the nanoantenna at any frequency *ω*. Hence, we use the polarization model in Eq. (22) for the nanoantenna. We parenthetically note that using Eq. (22) it is easy to find the general limitations on the absorbing and scattering cross sections of the nanoantenna (see review^36^). The equivalent circuit of the nanoantenna is shown in Fig. 2(a) and it contains an IEMF 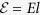 loaded by a series connection of the antenna radiation resistance *R*_rad_, the dissipation resistance *R*_dis_, capacitive impedance 1/*jωC* and inductive one *jωL*. This series connection corresponds to the Lorentzian model of the scatterer’s dispersion:


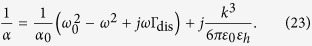


Comparing Eqs (22) and (23), we can relate the equivalent parameters with the corresponding parameters *α*_0_, *ω*_0_, and Γ_dis_ of the Lorentzian model:


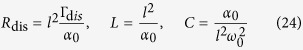


For the resonance frequency we have the usual expression 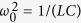.

**Figure 3:**
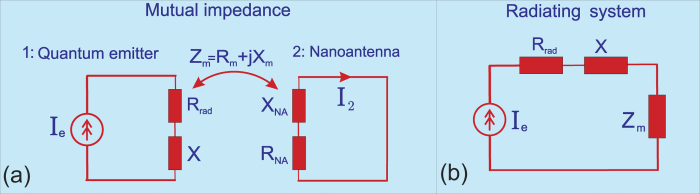
(**a**) Equivalent schemes of an optical emitter and a nanoantenna in terms of mutual impedance. (**b**) An equivalent scheme of an emitter with the mutual impedance added by the nanoantenna.

For the emitter we also start from the general circuit model Fig. 2(a). This equivalent circuit is a revision of the previously suggested scheme in^19^ (Fig. 3(a)). As an approximation of weak coupling, the electric dipole moment **d**_1_ = *d*_1_**z**_0_ is fixed at any frequency corresponding to the emission spectrum. Since the dipole moment is related to the effective current of the emitter *I*_*e*_ = *jωd*_1_/*l*_1_, the equivalent circuit in Fig. 2(b) comprising both emitter and nanoantenna is driven by a fixed current source *I*_1_ 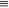 *I*_*e*_. The validity of the replacement of the circuit driven by the EMF 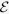 in Fig. 2(a) by the circuit shown in Fig. 2(b) which is driven by the current generator is ensured by the well-known equivalent generator theorem. In Fig. 2(b), we neglect the dissipation in the quantum source since the main mechanism of the decay rate is radiative 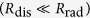, as mentioned previously. Here, for simplicity of notations, *R*_rad_ denotes the proper radiation resistance of the emitter denoted above as *R*_0,rad_.

The IEMF describing the mutual coupling of nanoobjects “1” and “2” in Fig. 2(b) can be replaced by mutual impedance *Z*_*m*_ and the real part of *Z*_*m*_ comprises an additional radiation resistance arising in the emitter and responsible for the Purcell factor. The corresponding modification of the equivalent scheme from the mutually induced EMF to the mutual impedance can be explained as follows: the emitter induces the IEMF 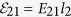 in nanoantenna “2”, where the field *E*_21_ is that produced by the emitter at the center of the nanoantenna *r*_2_, thus, this field can be written in form **E**_21_ = *z*_0_*A*_*ee*_*d*_1_, where *A*_*ee*_ is the electric field of an unit electric dipole with the origin at **r**_1_ 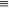 **r**_*d*_ evaluated at **r**_2_. In the case of symmetric mutual location of objects “1” and “2” the quantity *A*_*ee*_ is scalar. This IEMF is related to the current induced in the nanoantenna as 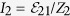, where *Z*_2_ is the impedance of the nanoantenna. The dipole moment of the latter 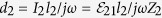 generates the scattered field *E*_12_ and the IEMF 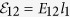 arises in the quantum emitter. Due to the reciprocity we can express **E**_12_ through the same coefficient *A*_*ee*_:


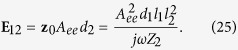


Since the current in the emitter is fixed, *I*_1_ = *jωd*_1_/*l*_1_ = *I*_*e*_, the IEMF 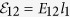 is equivalent to the mutual impedance 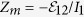 in accordance with the equivalent generator theorem. The minus sign in the relation 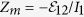 appears because the IEMF 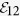 is directed opposite to the driving current *I*_1_ (Fig. 2(b)). Thus, in our final equivalent scheme as shown in Fig. 3(a), the IEMF 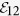 is replaced by the mutual impedance *Z*_*m*_ describing the contribution of the nanoantenna into the the emitter circuit. From the equivalent generator theorem and Eq. (25), we obtain


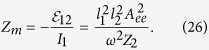


The final equivalent circuit of the radiating system where the presence of the nanoantenna is completely described by the mutual impedance *Z*_*m*_ is depicted in Fig. 3(b). The radiation of the whole system is created by the current generator *I*_1_ = *I*_*e*_ = *jωd*/*l*_1_ loaded by the series connection of the proper impedance *R*_rad_ + *jX* of the emitter and the mutual impedance *Z*_*m*_. In the reactance *X* of the emitter its proper *L*- and *C*-parameters are connected in series. In the mutual impedance *Z*_*m*_ the effective mutual inductance *L*_*m*_ and capacitance *C*_*m*_ are connected in parallel. It is imperative to explain this particular contrast.

The input impedance of the nanoantenna is a series connection of resistance *R*_2_, inductance *L*_2_, and capacitance *C*_2_. Values of *R*_2_, *L*_2_, and *C*_2_ can be found from the Lorentzian model of the nanoantenna Eq. (24). Substituting *Z*_2_ = *R*_2_ + *jωL*_2_ + 1/*jωC*_2_, denoting 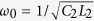, and assuming that *ω* ≈ *ω*_0_, Eq. (26) can be rewritten as


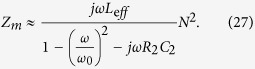


It is the standard formula in circuit theory that describes the impedance of a voltage transformer loaded by a low-loss parallel circuit with resonance frequency at *ω*_0_. In this formula *L*_e*ff*_ = *C*_2_*μ*_0_/*ε*_0_ is the effective inductance of the parallel circuit and the dimensionless value 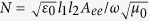 is an effective transformer parameter (called turns’ ratio in the electrical engineering). In the vicinity of the resonance the dispersion of *Z*_*m*_ is mainly determined by the denominator and we may neglect the frequency dependence of the effective transformer putting in Eq. (27) 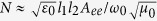. Then Eq. (27) describes the impedance of a parallel circuit with mutual inductance *L*_*m*_ = *μ*_0_*C*_2_*N*^2^/*ε*_0_ and mutual capacitance *C*_*m*_ = *ε*_0_*L*_2_/*N*^2^*μ*_0_ connected to effective resistors which are responsible for the mutual resistance *R*_*m*_.

The value *R*_*m*_ 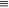 *R*_12_—the real part of the right-hand side of Eq. (27) comprises both radiative and dissipative resistance added to that of the emitter due to the presence of a nanoantenna. In the quasi-static approximation, the value of *A*_*ee*_ is real. Then *N* becomes real and positive that results in the Purcell effect larger than unity. If 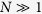 the Purcell factor at the resonance frequency may take much larger values.

Since the driving current is fixed, the power delivered by the emitter to its environment is equal to 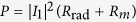. The Purcell factor in accordance with Eq. (16) takes the form


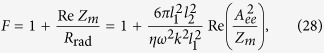


where we have used Eq. (15) for *R*_rad_ and substituted into Eq. (26). Now, applying the model of a Lorentzian scatterer to the nanoantenna, we can express the impedance *Z*_2_ of the nanoantenna through its polarizability *α*_2_ 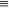 *α*_*NA*_. Thus, 
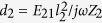
 and *α*_2_ = *d*_2_/*E*_21_. Therefore, Eq. (28) can be rewritten as


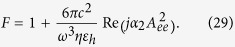


This expression clearly shows that the Purcell factor does not depend on the emitter “1” rather, depends only on the nanoantenna “2” and their mutual location. Therefore we speak about the Purcell factor *of an object* at a point with radius vector **r**_1_ – **r**_2_ with respect to the object. This factor is applicable to an arbitrary dipole emitter located at this point.

Now, we make some remarks on the above theoretical treatment. First, the problem of mutual coupling which we have solved above corresponds to the steady regime and is self-consistent at all frequency regimes. The Purcell factor has the physical meaning at frequencies close to *ω*_0_, since the emission has a finite decay rate and its spectrum has nonzero bandwidth. Second, our analysis remains valid in the case when the nanoantenna “2” has the resonance frequency *ω*_02_, which is different from the emission peak frequency of the quantum emitter, *ω*_0_ 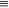 *ω*_01_. Despite this aforementioned situation, Eq. (29) holds and the equivalent scheme depicted in Fig. 3 remains valid, however the mutual impedance *Z*_*m*_ is not anymore the same parameter of a simple parallel circuit connected through the transformer. However, if the difference between *ω*_0_ and *ω*_20_ is large, Im *α*_2_ becomes very small at the emission frequency *ω*_0_, and the Purcell factor is close to unity.

The second remark is the more important one. In fact, the factor *A*_*ee*_ (electric field of a unit dipole with origin at **r**_1_ evaluated at **r**_2_) is complex due to the retardation effect. The imaginary part is relevant for calculation of the Purcell factor at the frequencies different from the resonance frequency of object “2”. Moreover, it is not exactly determined by the field of a unit dipole at the geometric center of the nanoantenna **r**_2*g*_. The electromotive force induced by a point emitter in nanoantenna “2” may be found accurately via the integration of the local field *E*_21_(**r**) over the volume of the nanoantenna. If the local field is strongly asymmetric with respect to its geometric center, the effective center *r*_2_ of the nanoantenna shifts from the point *r*_2*g*_ towards the emitter. Moreover, from the classical antenna theory^27^ it is known that for two-element array of dipole antennas, the mutual resistance is positive only when the antennas are collinear. This mutual location of dipoles “1” and “2” corresponds to Fig. 3(a) when the dipole moment of the emitter is stretched radially towards a plasmonic nanosphere. In the case of small distances *G* between the emitter and the sphere, we may approximate Im *A*_*ee*_ = 0 and *A*_*ee*_ ≈ 1/2*πε*_0_*ε*_*h*_*D*^3^, where *D *= *a *+ *G*. In accordance with Eq. (29) this results in a Purcell factor higher than unity. However, if the dipole is located so that their dipole moments are parallel and not shifted with respect to the nanosphere, the dipoles interact destructively. In this particular case, one cannot neglect Im*A*_*ee*_, moreover, its contribution for distances *D* comparable with *l*_1_ and *l*_2_ significantly exceeds that of the real part^27^. Then the second term in Eq. (29) becomes negative and makes the Purcell factor smaller than unity. This corresponds to the well-known situation: the mutual resistance *R*_*m*_ of a transmitting dipole and a closely located reflector antenna is negative, and the enhancement of the directivity is accompanied by the decrease of the efficiency^27^. For this case, the antenna theory provides, 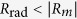
27. The energy balance is preserved and we have *F *> 0.

### Validation of the equivalent circuit

**Figure 4:**
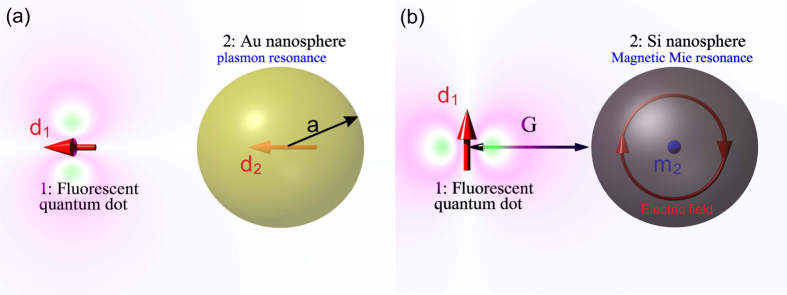
(**a**) A fluorescent emitter over a plasmonic (e.g. gold or silver) nanosphere has *F* > 1 when its dipole moment is radially directed since in this case *A*_*ee*_ > 0. (**b**) The same emitter over a dielectric (e.g. silicon) nanosphere has *F* > 1 when its dipole moment is azimuthal: in this case *A*_*em*_ > 0.

To validate our circuit model we apply it to an explicit structure depicted in Fig. 4(a). First, let us demonstrate that Eq. (29) based on the equivalent circuit fits the well-known analytical solution^37^. In Ref. 37, the Purcell factor has been calculated using the exact solution of the electrodynamic problem of a dipole radiating at the presence of a sphere of arbitrary radius *a* filled by an isotropic material of (generally complex) permittivity *ε*_*s*_. Eq. (6) of that particular paper refers to the radial polarization of the dipole and its location outside the sphere. The first term of the series corresponds to the dipole polarization of the sphere, thus for optically small spheres we can neglect the other terms.

In the framework of this approximate formula for the *radiative* Purcell factor^37^ reads:


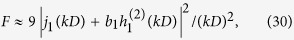


where *j*_1_(*X*) and 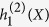 are, respectively, spherical Bessel function and Hankel function of second kind with order *n* = 1, *D* = *a* + *G* is the distance between the emitter and sphere centers, 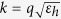 is the wave number in the host medium and coefficient *b*_1_ is given by formula^37^:


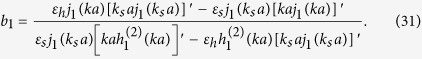


Here 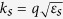 is the wave number inside the sphere. The dipole approximation is valid when 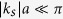, however, in reality when 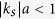. To compare the radiative Purcell factor in Eq. (30) with our result in Eq. (29) we have to remove losses that automatically equates the total Purcell factor to the radiative one. Therefore, we assume that *ε*_*s*_ is real. Then *k*_*s*_ is either real (if *ε*_*s*_ > 0) or imaginary (if *ε*_*s*_ < 0). In both of these cases the following approximations are valid for the spherical functions in Eq. (31):


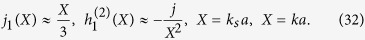


With these substitutions, the differentiation in Eq. (31) becomes elementary, and we obtain for *b*_1_ the result *b*_1_ = *jB*, where *B* is a real quantity:


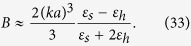


We restrict our analysis to the case 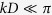. Then, the asymptotic relations in Eq. (32) are suitable for *X *= *kD*. Substituting expressions Eqs (32) and (33) in Eq. (30), we obtain:


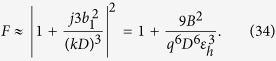


Substitution of Eq. (33) into Eq. (34) results in


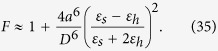


Our circuit model resulted in Eq. (29) which can be rewritten as


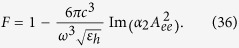


The quasi-static approximation for *A*_*ee*_ has been already introduced via


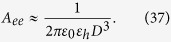


Since the sphere is lossless, from Eq. (23), we can infer


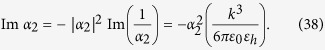


The quasi-static polarizability *α*_Q*S*_ of a small sphere is well-known (see Ref. 38), and we have:


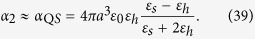


Substituting Eqs (37), (38) and (39) into Eq. (36) we obtain Eq. (35). Thus, the strict electrodynamic model and the present circuit model coincide within the framework of the dipole approximation.

**Figure 5:**
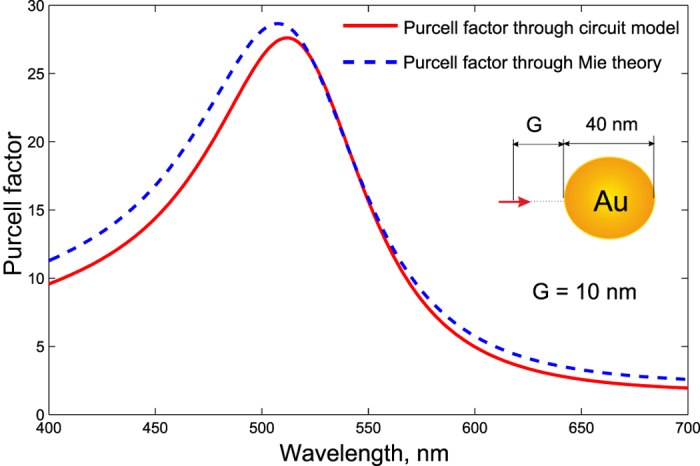
The radial Purcell factor of a golden nanosphere of diameter 40 nm at the distance *G *= 10 nm in air: our circuit model (red solid curve) and exact Mie theory (blue dashed curve). Values of the permittivity are taken from the experiments of Johnson and Christy^39^,^40^.

In order to validate our circuit model for a more interesting case where the sphere is resonant (plasmonic nanoantenna), we consider an explicit structure of a gold sphere of diameter 2*a* = 40 nm. The radially polarized emitter is located at the distance *G* = 10 nm from its surface. Values of the permittivity are taken from the experiments of Johnson and Christy^39^,^40^. The radiating system is located in the air, thus *ε*_*h*_ = 1. In Fig. 5, we present our calculation of the Purcell factor performed using Eq. (36) in comparison with the Mie theory. In our calculation we have complemented Eq. (39) by radiation losses in accordance with Eq. (22):


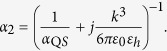


For the plasmon resonance band, our model is in agreement with the exact calculation. Our rough approximation for *A*_*ee*_ works in this band because at the resonance the dipole eigenmode is realized. The excitation mechanism is not very important, and the sphere is polarized by an emitter as if it were excited by a plane wave—nearly uniformly. The model becomes less accurate beyond the resonant band, where strong non-uniformity of the external field *E*_21_ implies strong non-uniformity of the polarization decaying versus the distance from the emitter. Due to this decay, the origin **r**_2_ of the dipole **d**_2_ shifts towards the emitter, and the effective distance decreases compared to *D* and *A*_*ee*_ increases compared to Eq. (37). Therefore, it is not surprising that our model utilizing the simple approximation Eq. (37) underestimates the Purcell effect at low frequencies.

### Extension of the circuit model

Next, we extend the equivalent scheme and generalize the Eq. (29). First, we observe that the equivalent circuit remains valid provided the nanoantenna is a magnetic dipole. Of course, we mean artificial magnetism when the vortex polarization currents in the subwavelength particle result in its magnetic dipole moment. Qualitatively, this insight is applicable, for example, to a submicron silicon sphere at its magnetic Mie resonance (see Refs 41, 42, 43). In Fig. 4(b), we have depicted the corresponding radiating system. The *z*-directed electric dipole *d*_1_ of the emitter 1 induces a magnetic dipole **m**_2_ = **x**_0_*m*_2_ at the center of the nanosphere “2”, which is related to the local magnetic field via the magnetic polarizability *β*_2_ 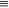 *m*_2_/*H*_*x*_. In this definition, *H*_*x*_ is the local field acting on the magnetic dipole. Here *H*_*x*_ 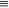 *H*_21_ is the magnetic field produced by the electric dipole **d**_1_ at the plane *z* = 0 at the distance *D*. It can be written as *H*_21_ = *jωA*_*em*_*d*_1_, where *A*_*em*_ ≈ 1/4*πD*^2^, in the quasi-static limit.

The magnetic dipole antenna can be modeled as an optically small loop with an effective area *S* and effective electric loop current *I* which is considered uniform around the loop. The magnetic dipole moment **m** = *μ*_0_*SI***n**, where **n** is a unit vector normal to the loop plane. The input impedance of the effective loop antenna equals to the ratio of IEMF 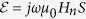 (where *H*_*n*_ is the normal component of the local magnetic field) to the electric loop current *I*. Hence, in the present case, the IEMF for the magnetic nanoantenna 2 resulting in the magnetic moment **m**_2_ is equal to 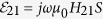, where *S* is the effective area of the polarization current loop of the nanosphere (it will cancel out eventually). The induced magnetic moment 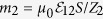 comprises the factor *ω*^2^ in the magnetic analogue of Eq. (21):


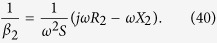


Note that, the Lorentzian model of the magnetic polarizability of a scatterer differs from the model of the electric polarizability by the factor *ω*^2^ (see e.g. in^41^). The analogue of Eq. (23) takes the form:


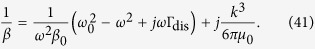


All other formulas of the Lorentzian model remain unchanged.

Accordingly, the equivalent circuit remains applicable. Magnetic moment *m*_2_ produces the electric field *E*_12_ = *jωA*_*em*_*m*_2_, by reciprocity theorem, the *A*_*em*_ is the same quantity as in the expression of *H*_21_. The corresponding IEMF 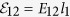 is recalculated into the mutual impedance in the same manner as above. Reproducing the same steps as for the electric dipole nanoantenna we obtain the expression for parallel-circuit, Eq. (27) for the *Z*_*m*_ with substitution of *N* = *ωS*_2_*l*_1_*A*_*em*_/*c* for the transformer parameter. For the Purcell factor we obtain an analogue of Eq. (29) in the form:


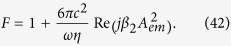


If the response of the nanoantenna comprises both electric and magnetic dipoles, each of these modes is described by its own equivalent scheme. If these dipole moments resonate at the same frequency, then both equivalent circuits are similar and can be unified. A more complicated equivalent scheme would correspond to different resonances of the electric and magnetic modes. However, it is important to notice that both the electric and magnetic modes obviously contribute into the total mutual impedance, and both these contributions can be constructive. So, the excitation of an additional mode in the nanoantenna may increase *R*_rad_, thus enhancing *F*. The same refers to higher multipoles of the nanoantenna: each of the multipole modes contributes into total *Z*_*m*_, and the coinciding or closely located resonances of high-order multipoles may result in huge values of the Purcell factor.

### Theoretical verification of the general approach

First, let us notice that our general approach resulted in Eq. (16)—an alternative to the conventional methods of calculating the Purcell factor. Although various numerical methods to solve the problems of nanophotonics and metamaterials have become widespread^44^, direct numerical calculation of the Purcell factor using Green’s function technique in Eq. (20) or scattered field technique in Eq. (1) faces fundamental difficulties. Indeed, the exact calculation of the microscopic field inside the quantum emitter as well as the exact calculation of the Green’s function at this point is challenging and time-consuming. Next, as shown in Ref. 45 another known method of the Purcell factor calculation through the volume and quality factor of the cavity mode (see the works^3^,^46^,^47^) provides a strong disagreement with the accurate theoretical model in Eq. (1), especially for plasmonic nanostructures. In finite systems and systems without losses, the method of integrating the radiated power flow through some spherical surface surrounding the radiating system has become popular. However, in structures with losses this method depends on the choice of the integrating sphere (even low losses may strongly deviate the result since the integration surface is very large). Finally, all these methods can not be realized experimentally and extended to the radio frequency range (which is one of the purposes of the present study).

In commercial software packages, such as CST Studio, a point dipole can be modeled as an optically very short dipole of a perfectly conducting wire excited by an ideal current source. Since it has a finite length *l*_1_, this dipole “1” in free space has a certain finite impedance, with real part *R*_0,in_ as radiation resistance. In presence of an arbitrary object “2” the IEMF 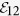 arises in the dipole and its input resistance modifies 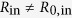. The input impedance results from exact simulations with the use of any reliable commercial software. The result for the Purcell factor *F* = *R*_in_/*R*_0,in_ should not depend on the length *l*_1_ of the equivalent Hertzian dipole. This method appears to be very practical and convenient for nanooptics. Moreover, it is more universal than all the aforementioned methods, because it is equally applicable to systems with or without losses.

**Figure 6:**
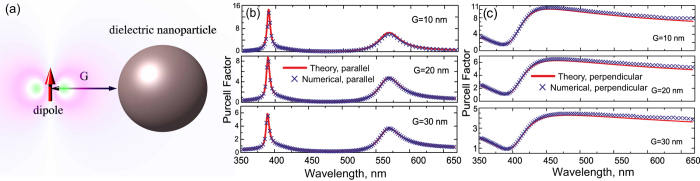
The Purcell factor extraction through a change of the input impedance in optics. (**a**) Illustration of a point dipole source located close to the dielectric spherical nanoparticle of the radius *a *= 70 nm. (**b**,**c**) Purcell factor dependence on the emission wavelength for the parallel (**b**) and perpendicular (**c**) dipole orientation with respect to the sphere.

To validate the general formula Eq. (16) we have studied the structure depicted in Fig. 4(b). In Fig. 6(a), the geometry of the problem under consideration is recalled. The quantum source is modeled as a Hertzian dipole of length 10 nm. The dielectric spherical nanoparticle of radius *a *= 70 nm and relative permittivity 15 is located at distance *G* from the dipole. The Purcell factor retrieved from numerical simulations as *F* = *R*_in_/*R*_0,in_ is compared with the exact solution^37^ in which now the series has been accurately evaluated. We studied both parallel and orthogonal dipole orientations, corresponding to Fig. 6(b,c), respectively, for three values of *G*. The exact solution and our results are in excellent agreement. For the orthogonal orientation at wavelength *λ* ≈ 570 nm the sphere experiences the magnetic Mie resonance, and at *λ* ≈ 390 nm—the electric dipole and magnetic quadrupole (makes highest contribution) Mie resonances. Unfortunately, our simplistic model resulting in Eqs (29) and (42) does not offer enough numerical accuracy because of two factors. First, the electric dipole mode cannot be neglected at the magnetic resonance. Second, the electric resonance holds at higher frequency, where the electromagnetic response of the nanosphere is not purely dipolar. However, for our current purpose it is enough that the exact version of our method—Eq. (16)—provides an excellent accuracy.

### Purcell factor for RF antennas

**Figure 7:**
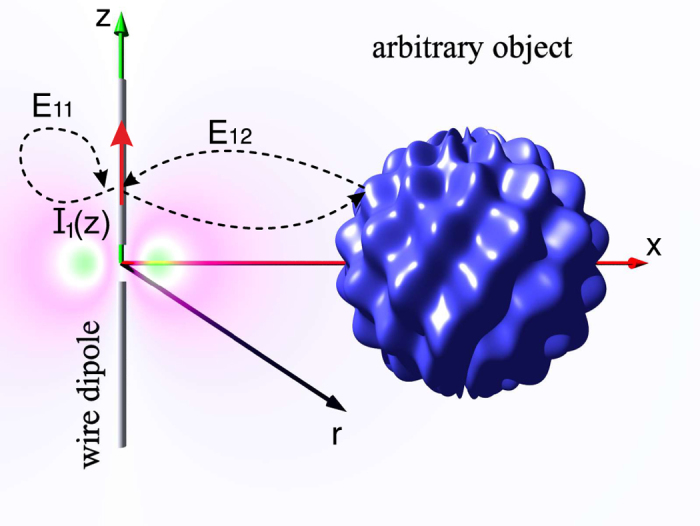
A schematic illustration of the radiative dipole antenna placed near an arbitrary scattering object. Each current density element of the antenna interacts with itself and other elements of the current (**E**_11_), as well as with an object (**E**_12_).

Now, let us go beyond the optical frequency range and extend the whole concept to radio frequencies; including microwaves, millimeter waves, and teraherz frequency ranges. Instead of a quantum emitter let us consider a dipole antenna 1 interacting with an arbitrary object 2, as it is sketched in Fig. 7. If the dipole 1 is resonant, e.g. has length *l*_1_ = λ/2 it can be excited by a short pulse (an analogue of the optical pumping) and will irradiate its energy at its resonant frequency *ω*_0_ during the finite emission time 1/γ_0_. If the radiation quality of the antenna is high, the time 1/γ_0_ is very long in terms of the period 2*π*/*ω*_0_. It may be reduced to 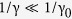 if an object 2 is located in the vicinity of the antenna 1 which increases its radiation resistance. Object 2 is not obviously a resonator tuned to the same frequency as it is adopted in optical applications of the Purcell effect. In accordance to our consideration in previous subsection it can be an arbitrary object constructively interacting with the antenna. Then the general equivalent circuit shown in Fig. 2(b) remains valid and results in the mutual impedance *Z*_*m*_. Of course, in the general case the mutual impedance is not obviously that of a parallel *RLC*-circuit. What is essential that Re*Z*_*m*_ 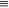 *R*_12_ should be positive and increase the input resistance of antenna 1. The impressed current *I*_*e*_ 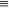 *I*_1_ is then determined by the dipole moment of the antenna 1 at the moment when the external pulse ends. In fact, this consideration and the representation of the object 2 via the mutual impedance *Z*_*m*_ have been known in antenna engineering for a long time, see Ref. 48.

It is difficult to significantly increase the radiation resistance of an already efficient antenna—that with the resonant length *l*_1_ = λ/2. Absolute values of *R*_*m*_ may be noticeable in this case, however the relative contribution will be modest. The concept of the Purcell factor becomes relevant for a short dipole—that with a low radiation resistance *R*_0,rad_, much lower than the internal resistance of the voltage generator applied to the radio antenna. As a rule, this is the output resistance of the feeding transmission line which usually takes the form *R*_out_ = 50 Ohms. If 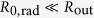, the presence of a low-loss object contributing positive mutual resistance may lead to much better impedance matching of the effective generator to the antenna and results in a higher radiation. At first glance, this radiation gain has nothing to do with the Purcell factor, which describes the emission regime. However, for a very short dipole 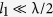 these values equate to one another.

The proper reactance of a short dipole antenna is capacitive. The spontaneous emission (quasi-harmonic radiation after a short pulse) is possible if the output impedance of the feeding line has the inductive reactance connected in series with *R*_out_. Then, in the absence of object 2, the emission is still described by the current source *I*_1_, at the frequency 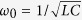 loaded by the resistance of the feeding line *R*_out_ = 50 Ohm and the radiation resistance 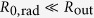. Most part of the energy is lost in *R*_out_ and only a small portion of the pulse energy is irradiated. The presence of object 2 changes this distribution increasing the radiation resistance and the decay rate by the factor *F* = *R*_rad_/*R*_0,rad_.

In the regime of the usual transmission at the frequency *ω*_0_, the steady-state voltage *V* at the output of the feeding line is loaded by the resistance *R*_out_ = 50 Ohm and the antenna input impedance *Z*_in_. The input impedance of a small antenna consists of a small radiation resistance 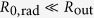 and a very high reactance *X*. In this case, the current 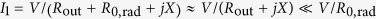 and only a small portion of the supplied power is radiated. The power is mainly reflected from the antenna back to the generator. The presence of object 2 increases *R*_r*ad*_, i.e. improves the matching of the antenna to the feeding line. The radiated power increases in accordance to formula *P*_rad_ = |*I*_1_|^2^*R*_rad_. However, matching of impedance remains poor since 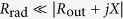. Therefore, we can write *I*_1_ = *V*/(*R*_out_ + *R*_rad_ + *jX*) ≈ *V*/(*R*_out_ + *jX*). The radiating current does not change in the presence of object 2 though the input resistance of the antenna 1 changes! The increase of the radiated power is solely described by the increase of the radiation resistance. Therefore, the gain in the transmitted radiation is equal to the Purcell factor *F* = *R*_rad_/*R*_0,rad_.

Briefly, for a very poor transmitting antenna 1 we may find the Purcell factor of object 2 from the usual radiation gain of the same antenna 1 in presence of the object 2. This factor describes the emission of the pulse energy by antenna 1 in presence of the radiation-enhancing object. It does not depend on the antenna itself and is fully determined by the properties of the object and its location. Following the same train of arguments, we can predict how much the antenna will radiate due to the presence of the object 2 if this antenna is tuned into resonance, excited in the absence and presence of the object by a pulse voltage, and find the decay rate of its emission after the pulse has traveled. We should stress that the Purcell factor of object 2 measured with the use of an antenna is not the same as the radiation enhancement of this antenna in the presence of object 2. Only in a special case when the probe antenna is a very poor emitter, they are approximately equal. The observation of this equivalence dramatically extends the relevance of the notion of Purcell factor to different areas.

Our last extension concerns the Purcell factor of an arbitrary object acting on a magnetic dipole antenna. We have already stressed that the magnetic dipole antenna is an optically small loop (can be multi-turn^27^) with an effective area *S* and electric current *I* which is practically uniform around the loop. The magnetic dipole moment **m***** ***= *μ*_0_*SI***n**, where **n** is a unit vector to the loop plane is related to the effective magnetic current as 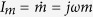. The input impedance of the loop antenna equals to the ratio of IEMF 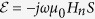 (where *H*_*n*_ is the normal component of the local magnetic field) to the induced electric current *I* and can be rewritten as *Z*_in,m_ = *H*_*n*_/*I*_m_. This offers a full analogy with the electric dipole antenna and corresponds to the duality principle. It is clear that the input impedance of the magnetic antenna is related to the Green’s function at the magnetic dipole origin as *G*_*zz*_(0, 0, *ω*) = −*jωZ*_in,m_/*q*^2^. After extracting the imaginary part from the last expression, we obtain the Purcell factor in the form of Eq. (16). So, all the theory developed above including the equivalent circuits remains valid.

### Measurement of the Purcell factor for microwaves

Now we demonstrate our method in experiment, retrieving the Purcell factor from measured input resistance of a radio antenna using Eq. (16). The input impedance of an antenna can be easily determined from the *S*-parameters. Namely, for a dipole antenna connected to a one-mode waveguide (e.g. a coaxial cable), the quantity *R*_in_ is related to the reflection coefficient *S*_11_ measured at the waveguide input and the characteristic impedance of the waveguide *Z*_w_^27^:


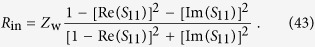


**Figure 8:**
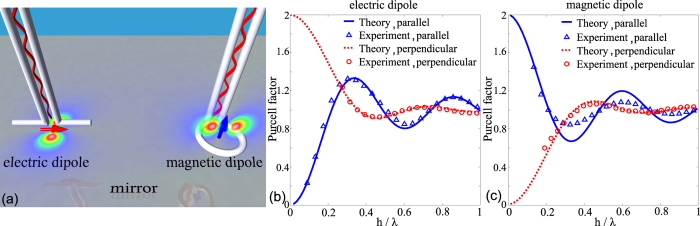
(**a**) Geometry of the experiment to measure the value of the Purcell factor for an electric dipole antenna near a perfect metallic mirror. (**b**) Measured results for the Purcell factor (symbols) along with the analytical results Eq. (44) for parallel and perpendicular orientations of the electric dipole antenna with respect to the mirror. (**c**) The same results for the magnetic dipole antenna.

In our experimental verification of the technique object “2” is a flat copper plate of optically large size and the antenna “1” is located near its center. This plate in the microwave range emulates the perfectly conducting plane and the Purcell effect in this case may be referred to as a special case of spontaneous emission near an interface^49^,^50^,^51^,^52^,^53^,^54^,^55^,^56^,^57^,^58^,^59^,^60^. For the perfectly conducting interface a simple analytical result for the Purcell factor was obtained in^51^,^61^. The expression for the electric (*F*_e_) and magnetic (*F*_m_) Purcell factor for either parallel 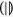 or perpendicular 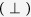 orientations are as follows:


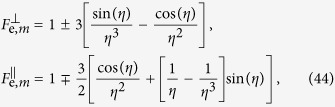


where *η* = 2*qh*, *h* is the height of the (electric or magnetic) dipole above the metal. The upper sign corresponds to an electric dipole, the lower to the magnetic one. We have compared the predictions of Eq. 44 with *F *= *R*_in_/*R*_0,in_. The experimental setup is schematically shown in Fig. 8. As two stems of an electric dipole antenna we use brass wires of length 0.4 cm soldered to the internal and external veins of the coaxial cable connected to a vector network analyzer. The wave impedance of the cable is equal to *Z*_w_ = 50 Ω that guarantees the regime 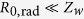. Magnetic dipole source is realized as a wire ring with the diameter 1 cm connected similarly. The measurement is performed in the spectral range 5–14 GHz which corresponds to wavelengths from 2.14 to 6 cm. The object 2 is a polished stainless steel sheet with sides 180 × 210 cm (the smallest mirror side greatly exceeds the largest wavelength and the diffraction effects are negligible). The antennas has been attached to an arm of a precise coordinate scanner which moved in the vertical directions, allowing us to measure the Purcell factor as a function of the emitter height. The main experimental results for electric and magnetic antennas are shown in Fig. 8b,c (squares and triangles correspond to two orientations of the electric and magnetic antennas). The solid blue and dashed red curves represent the theoretical values of the Purcell factor (44). Experimental and theoretical results are in excellent agreement. The Purcell factor exhibits oscillations with the period on the order of the wavelength when the source is moved vertically. These oscillations are due to the interference pattern which exhibits in the radiation resistance *R*_in_ ≈ *R*_rad_. It clearly indicates that our general Eq. (16) is applicable far beyond the quasi-static interaction between objects 1 and 2 assumed in the previous section. When *h* increases *F* eventually saturates at unity.

Slight disagreement can be noticed for the magnetic antenna. It is explained by a slight current inhomogeneity around the ring. This inhomogeneity appears when the magnetic dipole is parallel to the metal plane i.e. the loop is in the vertical plane. Definitely the lower half of the loop is stronger capacitively coupled to the metal plane than the upper one, and it results in this inhomogeneity. Notice, that for very small *h* we could not measure the Purcell factor due to the finite size of the our antennas when Eqs 44 become inapplicable. Comparing Figs 2 and 3 of^23^ with our Fig. 8 we have noticed that the Purcell factor of a mesoscopic (10 nm large) QD varies with respect to the distance to the silver mirror similarly to that of our radio antenna over the metal ground plane. Even deviations from Eq. (44) were also observed for a quantum dot, located too closely to the mirror^23^.

Importantly, in the microwave frequency range the electric dipole antenna is usually fed by a coaxial cable with non-negligible thickness. This factor results in the radiation from the cable open end and affects the measured Purcell factor. We directly measure not the input resistance *R*_in_ of the antenna but the sum of *R*_in_ and *δR*, where the last term is the radiation resistance of the open cable. Therefore, we have separately measured the input resistance of the open end of the cable which obviously equals to *δR* and subtracted it from *R*_in_ found with the use of Eq. (43). Otherwise, the disagreement in Fig. 8b,c would be more noticeable.

## Methods

### Numerical simulations

To validate our methodology of the Purcell factor extraction through input impedance of small sources in optics we have used the commercial software package CST Microwave Studio 2014. CST Microwave Studio is a 3D electromagnetic field solver based on finite—integral time domain (FITD) solution technique. A nonuniform mesh has been used to improve accuracy on the surface of spherical nanoparticle where the field concentration and variations are the greatest.

### Experimental technique

To measure the scattering parameters (S-parameters) the calibration method for vector network analyzer has been used. The method consists in determining the systematic error and exclusion of the errors by the mathematical correction of results. Studies were performed in both the frequency and time domains due to the built-Fourier transformation. The vector network analyzer that we have used is PNA E8362C. Measurements has been performed in an anechoic chamber.

## Additional Information

**How to cite this article**: Krasnok, A. E. *et al.* An antenna model for the Purcell effect. *Sci. Rep.* **5**, 12956; doi: 10.1038/srep12956 (2015).
